# Natural course of hemodynamically stable hemispheres contralateral to operated hemispheres in adult patients with ischemic moyamoya diseases

**DOI:** 10.1038/s41598-024-59141-0

**Published:** 2024-04-10

**Authors:** Young Sill Kang, Won-Sang Cho, Sun Mo Nam, Yuwhan Chung, Sung Ho Lee, Kangmin Kim, Hyun-Seung Kang, Jeong Eun Kim

**Affiliations:** Department of Neurosurgery, Seoul National University Hospital, Seoul National University College of Medicine, 101, Daehak-ro, Jongno-gu, Seoul, 03080 Republic of Korea

**Keywords:** Hemorrhage, Ischemia, Moyamoya disease, Natural course, Stroke, Cerebrovascular disorders, Stroke

## Abstract

The necessity of bilateral bypass in adult moyamoya disease (MMD) remains unclear despite its recommendation for pediatric and hemorrhagic cases. We aimed to investigate the natural course of hemodynamically stable unoperated hemispheres after bypass surgery for symptomatic and hemodynamically unstable hemispheres in adult patients with ischemic MMD. Among 288 patients, the mean age at the first operation of the unstable hemispheres was 40.8 ± 12.2 years. The mean follow-up period was 62.9 ± 46.5 months. 45 patients (15.6%) experienced stroke events in the unoperated hemisphere, consisting of hemorrhagic stroke in 8 (2.8%) and ischemic stroke in 37 (12.8%), including progressive transient ischemic attack in 25 (8.7%) and infarction in 12 (4.2%). Among them, 39 patients (13.5%) underwent bypass surgery. The annual risk of total stroke is 3.0%/patient-year, with 2.5% for ischemic stroke and 0.5% for hemorrhagic stroke. The 5- and 10-year cumulative risks of ischemic stroke were 13.4% and 18.3%, respectively, and those of hemorrhagic stroke were each 3.2%. The natural course of hemodynamically stable hemispheres contralateral to the operated ones appeared fairly good. Additional bypass surgery on the unoperated hemispheres should be considered for symptomatic and hemodynamically unstable hemispheres in adult patients with ischemic MMD during the follow-up.

## Introduction

Moyamoya disease (MMD) is characterized by steno-occlusive changes in the internal carotid arteries (ICAs) or their terminal branches, and compensatory development of extensive small collateral vessels^1,2^. It occurs worldwide but mostly in East Asian countries, such as Korea, Japan and China^3^. Pediatric patients mostly present with ischemic symptoms, while adults often present with hemorrhagic or ischemic events^1,4^. Although the exact pathogenesis has not been clearly elucidated, bypass surgery is well known to be effective in preventing ischemic and selectively hemorrhagic MMD^5–7^.

Bypass surgery on bilateral hemispheres is generally recommended for pediatric MMD due to the nature of the developing brain and hemorrhagic adult MMD regardless of hemodynamic status^7,8^. However, there is a lack of evidence for mandatory bilateral bypass surgery in adult MMD^9^. In this manuscript, ‘bilateral bypass surgery’ refers to bypass surgery on both hemispheres affected by moyamoya vasculopathy. Although only one hemisphere presents with severe symptoms indicating bypass surgery, both hemispheres undergo bypass surgery due to the bilateral nature of moyamoya vasculopathy, regardless of the asymptomatic and stable presentation in the other hemisphere. Bypass surgery is considered only for symptomatic and hemodynamically unstable hemispheres in some institutions, including our institution^5,7,10^; however, bilateral bypass surgery is adopted in other institutions^11,12^. There is no detailed recommendation on the necessity of bilateral bypass surgery in ischemic MMD, even in some guidelines^9,13–15^. In this study, we aimed to investigate the natural course of hemodynamically stable hemispheres with asymptomatic or benign symptoms contralateral to the operated hemispheres in adult patients with ischemic MMD to gain insights to whether bilateral bypass surgery should be mandatory or selective.

## Methods

### Patient selection

**Figure 1:**
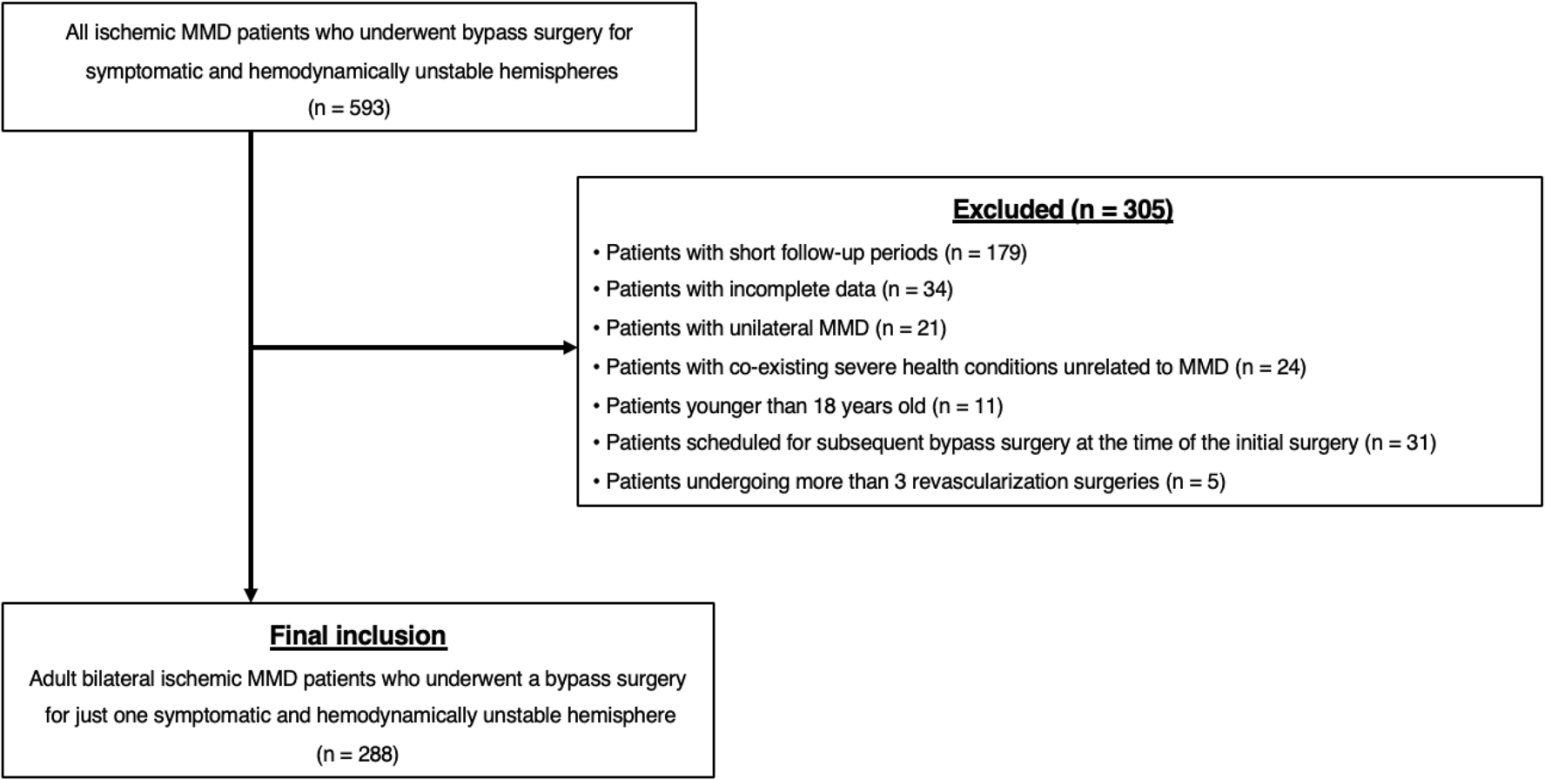
Flowchart of the patients. The medical records of 593 adult patients with bilateral ischemic MMD who had undergone one-sided bypass surgery in our institute between July 2004 and September 2021 were reviewed A total of 305 patients were excluded from the study due to failure to meet the inclusion criteria. Finally, a total of 288 hemispheres in 288 patients were included in the study.

Under the approval of the Institutional Review Board, we retrospectively reviewed the medical records of 593 patients with MMD who had undergone one-sided bypass surgery in our institute between July 2004 and September 2021. After the review of medical records, a total of 288 hemispheres in 288 patients were finally included (Fig. 1). The inclusion criteria were as follows: (1) age ≥ 18 years; (2) compatibility with the diagnostic guidelines^10^; (3) available medical records during the follow-up period; (4) combined bypass surgery for just one symptomatic and hemodynamically unstable hemisphere in patients with bilateral MMD; and (5) follow-up duration of 6 months or more. The follow-up started from the first bypass surgery of hemodynamically unstable and symptomatic hemispheres and ended when stroke occurred or until the last radiological imaging in the event-free cases. The baseline characteristics are presented in Table 1. The male to female ratio was approximately 1:2, and the mean age when the patients underwent the first bypass surgery was 40.8 ± 12.2 years old (range 18–71.3 years). The overall mean follow-up duration was 62.9 ± 46.5 months (range 6–200.7 months).

**Table 1:** Clinical characteristics.

Characteristic	Values
Total patients	288
Female sex	191 (66.3)
Age, years	40.8 ± 12.2 (18–71.3)
Concomitant medical diseases	
Hypertension	92 (31.9)
Hyperlipidemia	53 (18.4)
Diabetes mellitus	33 (11.5)
Thyroid dysfunction	9 (3.1)
Familial type	55 (19.1)
Suzuki grade	
Operated hemispheres	4.3 ± 0.7 (2–6)
Unoperated hemispheres	3.3 ± 1.1 (2–6)
Follow-up duration, months	62.9 ± 46.5 (6–200.7)

Values are shown as number of the patients (percent) or mean ± SD (range).

The most common initial clinical manifestations related to the unoperated hemispheres were nonspecific symptoms, including headache and dizziness, and no symptoms in 65% of the patients (Table 2). Repeatedly occurring same types of headaches, and dizziness with no clear origin in the ear, gastrointestinal and cardiovascular systems, were categorized as non-specific symptoms. Headache was assigned to the hemisphere which it occurred in isolation from. When headache and dizziness each were accompanied with specific neurological symptoms such as TIA, infarction, hemorrhage, seizure and involuntary movement, each of them was assigned to the hemisphere which was related to the specific symptoms. For isolated non-specific symptoms without clear laterality, headache and dizziness respectively were assigned into both hemispheres. Seizure laterality was confirmed with semiology and electroencephalographic findings. Seizure was secondary to the infarction and hemorrhage. Initial clinical manifestations related to unoperated hemispheres were divided into three groups: hemorrhagic, ischemic (encompassing both transient ischemic attacks (TIAs) and cerebral infarctions), and with other symptoms (characterized by nonspecific symptoms such as headache, dizziness, seizure, involuntary movement, and no symptoms). Cumulative stroke risks were measured.

**Table 2:** Initial clinical manifestations related to each hemisphere.

Clinical manifestations	Unoperated hemispheres	Operated hemispheres
Non-specific symptoms^†^	120 (41.7)	74 (25.7)
No symptoms	67 (23.3)	4 (1.4)
Ischemia		
Transient ischemic attack	74 (25.7)	198 (68.8)
Infarction	33 (11.5)	92 (31.9)
Hemorrhage	17 (5.9)	39 (13.5)
Seizure	10 (3.5)	7 (2.4)
Involuntary movement	3 (1.0)	2 (0.7)

Values are shown as number of the hemispheres (percent).

As initial clinical presentations of MMD are various and occur often simultaneously, multiple selections of the manifestation were allowed.

^†^Nonspecific symptoms include headache and dizziness.

This study was approved by the Seoul National University Hospital Institutional Review Board (approval number 2211-078-1378). All methods were performed in accordance with the relevant guidelines and regulations.

### Radiological and clinical evaluations

In all patients, bilateral MMDs were angiographically confirmed and graded using the Suzuki grade^2^. The mean Suzuki grades for the first operated and unoperated hemispheres were 4.3 ± 0.7 (range 2–6) and 3.3 ± 1.1 (range 2–6), respectively. Radiological evaluation and follow-up were performed based on computed tomography, single photo emission computed tomography and various magnetic resonance (MR) imaging techniques. SPECT imaging was performed using a triple-headed camera (Prism 3000, Picker International) with a low-energy high-resolution fanbeam collimator. Forty step-and-shoot images were acquired, with 3° intervals and 20 s per step. Technetium-99m hexamethyl propylenamine oxime (Tc-99 m-HMPAO) basal/acetazolamide brain perfusion SPECT was used^16^. Usually, follow-up imaging was performed at 6 months and every year for the first 3 years. Thereafter, MR imaging and/or SPECT were performed every 2 years. The interval is shortened to less than 2 years if there are symptom changes and lengthened to more than 2 years if patients remain stable.

The diagnosis of cerebral hemorrhage and infarction was confirmed by neuroradiologists. A normal or mild decrease in basal perfusion and a sustained reserve capacity less than a 50% decrease in basal perfusion under acetazolamide challenge were defined as a hemodynamically stable status^5,17^. The basal perfusion and reservoir capacity states were qualitatively evaluated by the nuclear medicine specialists. Initial clinical manifestations were defined as all the symptoms related to the unoperated hemispheres. Seizure and involuntary movement were differentiated based on electronic encephalographic findings and neurological examination. During the follow-up, both symptomatic and asymptomatic strokes (hemorrhagic or ischemic) in the unoperated hemispheres were all checked up as events. Ischemic strokes were defined as cerebral infarction and progressive TIA. The clinical status of the patients without events was measured using the Karnofsky Performance Scale (KPS)^18^ and the modified Rankin Scale (mRS)^19^ during the follow-up after subsequent surgery, when the unoperated hemisphere was eventually operated.

### Statistical analysis

Continuous variables are expressed as the mean ± SD (range). The annual risk of stroke was calculated with a person-year method. Cumulative stroke risk was measured using the Kaplan‒Meier method. Comparison of the risks between ischemic and hemorrhagic strokes was performed using log-rank tests. Stroke events were indicated on the Kaplan‒Meier curve as uncensored, which corresponded to endpoints of hemispheres with stroke events. The remaining endpoints were the last follow-up with both radiological and clinical check-ups, which were censored on the Kaplan‒Meier curve. The KPS and mRS of the patients without events at the beginning and end of the follow-up period were compared by paired t tests, as they were normally distributed. Differences with p values < 0.05 were considered statistically significant. All statistical analyses were performed using GraphPad Prism 9 software (GraphPad Software Inc., San Diego, CA, USA).

#### Informed consent to patients

Considering the retrospective design and anonymized data utilized, the local ethics committee of Seoul National University Hospital approved a waiver of informed consent requirements.

## Results

Of all the 288 included patients, 45 patients (15.6%) had stroke events in the unoperated hemispheres. Ischemic and hemorrhagic strokes occurred in 37 (12.8%) and 8 (2.8%) hemispheres, respectively. The ischemic stroke events included cerebral infarction, which occurred in 4.2% (n = 12) of the patients, and progressive TIA, which occurred in 8.7% (n = 25) (Table 3). Three patients had asymptomatic cerebral infarction. The annual risk of total stroke was 3.0%/person-year, consisting of those of ischemic and hemorrhagic strokes at 2.5 and 0.5%/person-year, respectively. In an additional analysis regarding the stroke location of stroke recurrence according to the dominancy of hemispheres, there were no differences between dominant and non-dominant hemispheres. Twenty-three events occurred in the dominant hemispheres and 22 occurred in the non-dominant ones. In the dominant hemispheres, there were 4 hemorrhagic strokes and 7 cerebral infarctions including 2 asymptomatic infarctions. In the non-dominant hemispheres, there were 4 hemorrhagic strokes and 5 infarctions including 1 asymptomatic one.

**Table 3:** Stroke event and its annual risk in unoperated hemispheres.

	Events	Annual risk
Total	45 (15.6)	3.0
Ischemic stroke	37 (12.8)	2.5
Infarction	12 (4.2)	0.8
Transient ischemic attack	25 (8.7)	1.7
Hemorrhagic stroke	8 (2.8)	0.5

Values are shown as number of the events (percent) or %/person-year.

Worsening of the TIA occurred in 12 dominant and 13 non-dominant hemispheres, respectively.

In the operated hemispheres, 12 stroke events (4.2%) occurred. Ischemic stroke occurred in 6 hemispheres (2.1%), including 4 infarcts (1.4%) and 2 TIAs (0.7%). A total of 6 hemorrhagic strokes (2.1%) were observed, including 3 cases of intracerebral hemorrhage with intraventricular hemorrhage, 2 cases of intracerebral hemorrhage and 1 case of intraventricular hemorrhage. The annual risk of total stroke in the operated hemispheres was 0.8% per person-year, with ischemic and hemorrhagic strokes accounting for 0.4%/person-year each.

The initial clinical manifestations of the unoperated hemispheres of the 8 patients who experienced hemorrhagic events were hemorrhage in 1, TIA in 3, infarction in 2, nonspecific symptoms in 2 and seizure in 1. Notably, multiple manifestations per individual were possible.

**Figure 2:**
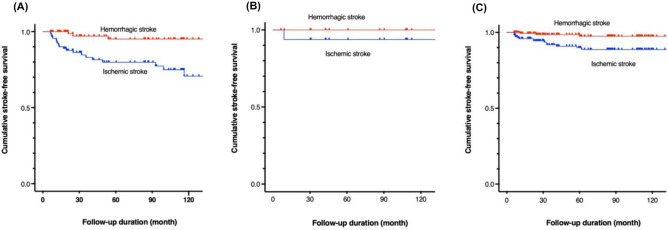
Kaplan‒Meier curves for ischemic and hemorrhagic strokes related to the initial clinical manifestations in the unoperated hemispheres. (**A**) Ischemic group (n = 93): the 5-year cumulative risk of ischemic stroke was 20.2%, increasing to 29.3% at 10 years. Hemorrhagic stroke risk remained at 4.9% for both periods. Ischemic stroke risk significantly higher than hemorrhagic stroke (p < 0.01). (**B**) Hemorrhagic group (n = 17): the 5- and 10-year cumulative risks of hemorrhagic stroke were both 0%, while those for ischemic stroke were both 6.3%. The survival curves of these groups did not show significant differences (p > 0.99). (**C**) Group with other symptoms (n = 180): 5- and 10-year cumulative risks of ischemic stroke were 10.1% and 11.3%, respectively. In contrast, the cumulative risks of hemorrhagic stroke were both 2.6%. Ischemic stroke risk was significantly higher than hemorrhagic stroke risk (p < 0.01).

We further analyzed the cumulative risks of stroke associated with the initial clinical manifestations in the unoperated hemispheres. We categorized these manifestations into three groups: ischemic (n = 93), hemorrhagic (n = 17), and other symptoms (n = 180). In the ischemic group, the 5- and 10-year cumulative risks of ischemic stroke were 20.2% and 29.3%, respectively, while the risk of hemorrhagic stroke remained consistent at 4.9% for both periods. Ischemic stroke risk was significantly higher than hemorrhagic stroke risk (p < 0.01). In the hemorrhagic group, the 5- and 10-year cumulative risks of hemorrhagic stroke were both 0%, while those for ischemic stroke were both 6.3%. The survival curves of these groups did not show significant differences (p > 0.99). In the group with other symptoms, the 5- and 10-year cumulative risks of ischemic stroke were 10.1% and 11.3%, respectively. In contrast, the 5- and 10-year cumulative risks of hemorrhagic stroke were both 2.6%. The cumulative risk of ischemic stroke was significantly higher than that of hemorrhagic stroke (p < 0.01). The cumulative risks of stroke associated with the initial clinical manifestations are presented visually in Fig. 2.

Among 45 patients who experienced subsequent stroke events, 39 patients (13.9%) finally underwent bypass surgery on the unoperated hemispheres. The mean time interval from the first operation to the contralateral hemisphere was 43.1 ± 48 months (range 6.9–208.8 months). The reasons to perform bypass surgery included progressive TIA in 25 hemispheres (64.1%), symptomatic cerebral infarction in 8 (20.5%), hemorrhagic stroke in 5 (12.8%), and asymptomatic cerebral infarction in 1 (2.6%). Of the remaining 6 patients, 3 with hemorrhagic stroke and 3 with cerebral infarction did not undergo bypass surgery. One patient with hemorrhagic stroke was observed without surgery because the moyamoya vessels were not definitively implicated. Another patient with hemorrhage refused the indicated bypass surgery. The third patient was too in a poor clinical condition to undergo the surgery. Two patients with cerebral infarction had an asymptomatic lacunar infarct in the occipital lobe and periventricular white matter, respectively. The other with cerebral infarction had a transiently symptomatic lacunar infarct in the occipital lobe. All the patients with cerebral infarction have not showed additional problems yet since then.

**Figure 3:**
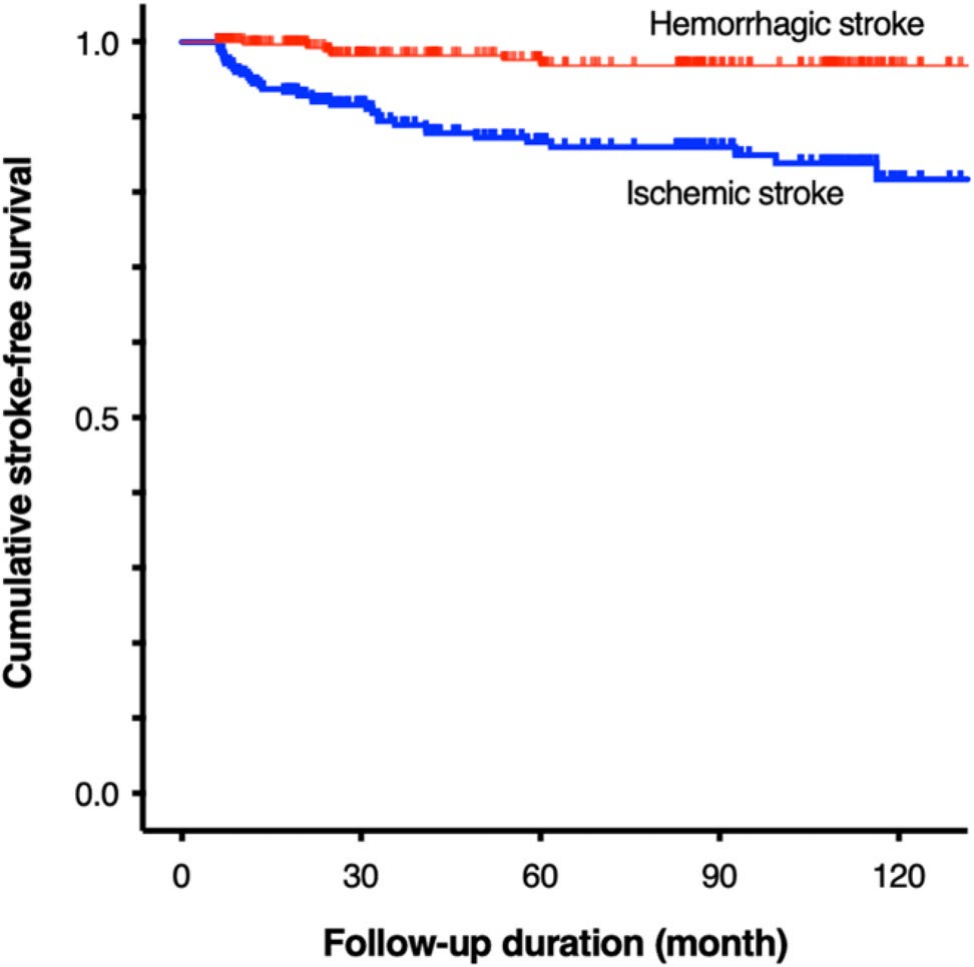
Kaplan‒Meier curves for ischemic and hemorrhagic stroke risks in the unoperated hemispheres during the follow-up. The 5- and 10-year cumulative risks of ischemic stroke were 13.4% and 18.3%, respectively. Those of hemorrhagic stroke were each 3.2%. The cumulative risk of ischemic stroke was significantly higher than that of hemorrhagic stroke (p < 0.01).

The 5- and 10-year cumulative risks of strokes in the unoperated hemispheres are shown in Fig. 3. The 5- and 10-year cumulative risks of ischemic stroke (TIA and infarction) were 13.4% (9.8% and 3.8%, respectively) and 18.3% (12.8% and 6.1%, respectively), respectively. The 5- and 10-year cumulative risks of hemorrhagic stroke were each 3.2%. The cumulative risk of ischemic stroke was significantly higher than that of hemorrhagic stroke (p < 0.01).

For the 243 patients (84.4%) with no significant events during the follow-up, the initial and last clinical KPS scores were 94.3 ± 8.4 (range 50–100) and 96.1 ± 8.8 (range 50–100), respectively. The mRS scores were 0.5 ± 0.8 (range 0–4) and 0.4 ± 0.8 (range 0–4), respectively. The clinical status at the end of follow-up, based on both the KPS and mRS scores, significantly improved (each p < 0.01). Analyzing the KPS and mRS scores in 39 patients who underwent subsequent bypass surgery, the KPS scores were 94.9 ± 10.0 (range 5–100) in the initial, 91.5 ± 20.5 (range 20–100) just before the surgery, and 94.1 ± 9.9 (range 60–100) in the first follow-up after the surgery, respectively. The mRS scores were 0.5 ± 0.8 (range 0–4), 0.6 ± 1.4 (range 0–5), and 0.6 ± 1.0 (range 0–4), respectively. There were no statistical significances in the KPS and mRS each among three periods. The time period between the initial and just before the surgery was 43.1 ± 48.0 months (range 5.3–17.5 months), and that between just before and the first follow-up after the surgery was 7.2 ± 43.3 months (range 6.8–208.7 months).

## Discussion

The present study was initiated by the question of whether bypass surgery for bilateral hemispheres would be inevitable in adult patients with ischemic MMD. In the current study, we retrospectively investigated hemodynamically stable unoperated hemispheres in 288 adult patients who underwent unilateral bypass surgery for symptomatic and hemodynamically unstable hemispheres. During the entire follow-up of 62.9 ± 46.5 months, 15.6% of all the patients experienced stroke events, and 13.5% eventually underwent bypass surgery for the remaining hemispheres. The annual risk of total stroke in the unoperated hemispheres was 3.0%/patient-year, including 2.5% for ischemic stroke and 0.5% for hemorrhagic stroke. The annual risk of stroke in the operated hemispheres was 0.8%/ person-year, with ischemic and hemorrhagic strokes accounting for 0.4%/person-year each. This finding is consistent with our previous study^5^. The estimated 5- and 10-year cumulative risks of ischemic stroke were 13.4% and 18.3%, respectively. Those of hemorrhagic stroke were each 3.2%. To the best of our knowledge, there is no study with a large number of patients reporting the natural course of unoperated hemispheres contralateral to operated hemispheres in adult patients with ischemic MMD. These outcomes are expected to be helpful in establishing more detailed guidelines for the surgical indications of ischemic MMD in adult patients.

Previous reports on the natural history of MMD included heterogeneous patient populations. Annual stroke rates varied, with 13.3% reported in a North American adult study^20^ and ~ 3.5% described as a benign course in a Finnish population^21^. The result of our study aligns more closely with the ~ 3.5% annual stroke rate described as a benign course in a Finnish population^21^. The overall cumulative 5-year risks of stroke varied from 40 to 82%^22–24^. On the other hand, the annual incidences of cerebrovascular events in asymptomatic MMD varied from 2.4 to 5.7%^25,26^. In our previous study with hemodynamically stable adult MMD, the annual risks of all strokes and ischemic stroke were 4.5% and 2.2%, respectively, and the 5- and 10-year risks of recurrent ischemia in the ischemic group were 13% and 28%, respectively^27^. Kuroda et al. reported a stroke risk of 3.2%/person-year in asymptomatic MMD^25^. Taken together with the results in this study, the natural course of the unoperated stable hemispheres seems similar to that of benign or asymptomatic groups, and better than that of symptomatic or general MMD patient groups. In addition to this study, subsequent bypass surgery for the patients who experienced clinical deterioration during the follow-up succeeded in preservation of their initial clinical status. And the other majority patients with no significant events during the follow-up showed significant improvement of clinical status at the last follow-up compared to that in the initial. Therefore, it seems reasonable to recommend selective bypass surgery rather than mandatory bilateral surgery, at least in adult patients with ischemic MMD with benign or asymptomatic hemispheres.

There have been reports about the effects of revascularized hemispheres on the unoperated contralateral hemispheres. Improved cerebrovascular reserve capacity and cerebral blood flow even in the unoperated hemispheres after unilateral bypass surgery were reported several times^28–31^. Our study reports an annual risk of stroke events in non-operated hemispheres of 3.0% per year, which is relatively low and similar to the reported range of 2.4% to 5.7% for asymptomatic MMD^25,26^. This potentially positive outcome raises the intriguing possibility that improved hemodynamic status on the operated side might favorably impact the unoperated hemisphere. Deckers et al. reported a tendency of decreased TIA frequency originating from the contralateral hemispheres^29^. They speculated that the hemodynamic improvement of the ipsilateral hemispheres due to unilateral bypass surgery decreases collateral demand from the contralateral hemispheres, resulting in subsequent improvement of hemodynamic status in the contralateral hemispheres^29^. In addition, an increase in cortical thickness in the contralateral nonoperated hemispheres was reported, even though it was smaller and less consistent^32^. This finding hints at the potential for revascularization to influence other biological processes beyond immediate hemodynamic shifts, such as structural changes. These previous studies suggest that bypass surgery can improve perfusion status in the contralateral hemispheres. However, it remains unclear whether improved hemodynamics in the unoperated hemispheres after the bypass surgery of contralateral hemispheres is sufficient to affect the clinical course of the unoperated hemisphere, which is consistent with current studies^25–27^. All the patients in this study showed improved perfusion status, along with the stroke-free survival in 84.4% of them during the follow-up. Due to the lack of a comparison group, it is difficult to determine whether improved hemodynamic status influenced stroke-free survival. Because of the retrospective nature of our study, establishing a control group was not feasible. However, given the rarity of MMD, our findings provide new insights into the natural course of unoperated hemispheres in patients who underwent surgery on one side. To ascertain the positive effect, further research with comparative studies with large-scale observational studies with longer follow-up periods is necessary.

This study has several limitations. First, its retrospective design introduces the potential for selection bias. Second, we did not use a case–control design, which is ideal to get a final answer. However, MMD is a rare disease that it was not easy even to collect the patients’ data alone. Third, the analysis lacks adjustment for potential confounding factors like age, initial hemodynamic status, clinical presentation, and other stroke risk factors. This omission could introduce bias into the results. In the future study, those variables will be incorporated into analysis to provide a better understanding of the natural clinical course of hemodynamically stable hemispheres contralateral to operated hemispheres. Fourth, the follow-up duration varied among patients, which could reduce the reliability of the event rates and cumulative risks. Fifth, asymptomatic lesions in this study were detected during the routine imaging follow-up, so the time point data may not indicate the exact time of lesion occurrence. Finally, SPECT is a somewhat subjective in evaluating the perfusion status, compared to the MR and CT. However, it was feasible to use SPECT data because they were available in all the patients.

## Conclusions

The natural course of hemodynamically stable hemispheres contralateral to the operated ones was favorable in adult patients with ischemic MMD. Selective bypass surgery is thought reasonable rather than mandatory bilateral bypass surgery. Surgical treatment should be considered only for symptomatic and hemodynamically unstable hemispheres. However, long-term follow-up is necessary for the adult patients with ischemic MMD who underwent unilateral bypass surgery as a small proportion of these patients may experience clinical deterioration over time, although uncommonly.

## Data Availability

The datasets used and/or analyzed during the current study are available from the corresponding author on reasonable request.
